# Bleeding Frequency and Its Association With Joint Health and Functional Independence in Hemophilia: A Cross-Sectional Study Using the Hemophilia Joint Health Score (HJHS) and Functional Independence Score in Hemophilia (FISH)

**DOI:** 10.7759/cureus.107298

**Published:** 2026-04-18

**Authors:** Piyush Malik, Sudhir K Atri, Vikas Chaudhary, Nitin Kumar

**Affiliations:** 1 Department of Medicine, Pandit Bhagwat Dayal Sharma Post Graduate Institute of Medical Sciences, Rohtak, IND

**Keywords:** bleeding frequency, functional independence, functional independence score in hemophilia, hemophilia, hemophilia joint health score, hemophilic arthropathy

## Abstract

Background and aim

Hemophilia is a genetic bleeding disorder defined by a deficiency of coagulation factor VIII or IX, which results in recurrent bleeding episodes that frequently involve the joints. Repeated hemarthrosis results in progressive joint damage and functional impairment. The Hemophilia Joint Health Score (HJHS) and Functional Independence Score in Hemophilia (FISH) are standardized tools used to assess joint status and functional ability in patients with hemophilia. This study aimed to assess joint health and functional independence in individuals with hemophilia using the HJHS and FISH and to determine the association between bleeding frequency, joint damage, and functional outcomes.

Methods

This cross-sectional study was carried out at a tertiary healthcare center in Northern India between December 2023 and December 2024. A total of 100 adult persons with hemophilia A or B were included. Joint health was evaluated using HJHS version 2.1, and functional independence was assessed using the FISH score. Annual bleeding frequency was assessed based on documented bleeding records. Associations between bleeding frequency, joint health, and functional status were analyzed.

Results

Among the participants, 90% had hemophilia A and 10% had hemophilia B, with 98% having severe disease. The knee was the most commonly affected joint. The mean HJHS score was 15.64 ± 8.84, and the mean FISH score was 29.07 ± 2.79. Higher bleeding frequency was significantly associated with increased HJHS scores and lower FISH scores (p < 0.001). A strong negative correlation was observed between HJHS and FISH scores (r = -0.905, p < 0.001).

Conclusions

Increasing bleeding frequency is associated with worsening joint health and reduced functional independence in persons with hemophilia. The combined use of HJHS and FISH provides a practical approach for assessing joint health and functional impairment and may assist clinicians in monitoring disease progression and guiding therapeutic interventions.

## Introduction

Hemophilia is an inherited bleeding disorder characterized by a deficiency of coagulation factor VIII (hemophilia A) or factor IX (hemophilia B), resulting in impaired hemostasis and recurrent bleeding episodes [1]. The disorder is characterized by an X-linked recessive inheritance pattern, mainly affecting males, while females are typically asymptomatic carriers [2]. Based on residual levels of clotting factors, hemophilia is divided into severe (<1% factor activity), moderate (1-5%), or mild (>5-40%) [3]. Patients with severe disease frequently experience spontaneous bleeding episodes, particularly involving the musculoskeletal system. The diagnosis and management of hemophilia are guided by internationally accepted recommendations, including the World Federation of Hemophilia 2020 guidelines [4].

Joint bleeding, termed hemarthrosis, is the leading clinical presentation of hemophilia and predominantly involves the musculoskeletal system, which accounts for approximately 80% of bleeding episodes [5,6]. Recurrent hemarthrosis triggers synovial inflammation, hypertrophy, and progressive cartilage degeneration, ultimately leading to chronic hemophilic arthropathy [7]. Over time, these pathological changes result in pain, joint deformity, reduced range of motion, and substantial impairment in physical functioning and quality of life [6]. Weight-bearing joints such as the knees, ankles, and elbows are particularly susceptible to repeated bleeding episodes due to mechanical stress and increased synovial vascularity [8].

The early identification and continuous monitoring of joint deterioration are vital to prevent permanent disability in patients with hemophilia. Several clinical scoring systems have been developed to objectively evaluate joint health and functional ability in patients with hemophilia. The Hemophilia Joint Health Score (HJHS), created by the International Prophylaxis Study Group, aims to identify early joint issues and evaluate the severity of hemophilic arthropathy via a standardized clinical evaluation of the elbows, knees, and ankles [9]. It remains one of the most widely used clinical instruments for evaluating joint damage and monitoring disease progression in individuals with hemophilia [10]. In addition to structural joint assessment, evaluation of functional ability is important for understanding the overall impact of the disease on daily living. The Functional Independence Score in Hemophilia (FISH) is a validated, performance-driven tool aimed at evaluating functional independence in activities such as self-care, transfers, and mobility [11].

Together, HJHS and FISH provide complementary information regarding structural joint damage and functional performance in individuals with hemophilia. Understanding the relationship between joint health and functional independence may help clinicians better evaluate disease burden and guide treatment strategies. The present study was therefore undertaken to assess joint health status and functional independence among persons with hemophilia attending a tertiary care center in Northern India using the HJHS version 2.1 and the FISH. We hypothesized that higher bleeding frequency would be associated with worse joint health and reduced functional independence.

## Materials and methods

Study design and setting

This cross-sectional study was carried out at a tertiary care facility in Northern India for a duration of 12 months, from December 2023 to December 2024.

Study population and eligibility criteria

A convenience sampling approach was adopted for this study. All eligible adult persons with hemophilia presenting to the hemophilia day care center and outpatient department during the study period were included using a consecutive sampling method. The sample size was determined based on feasibility and the number of eligible participants available during the study duration. A total of 100 participants were enrolled, which was considered adequate to allow meaningful statistical analysis and improve the robustness and reliability of the findings.

Eligible participants were aged 18 years or older and had confirmed hemophilia with factor VIII or IX levels less than 40% of normal. Patients receiving either prophylactic or on-demand factor replacement therapy were included in the study. Patients with acute musculoskeletal injury, hemarthrosis within the preceding three weeks, or recent trauma affecting the joints were excluded to avoid transient inflammatory changes that could influence joint scoring. Prior to enrollment, each subject provided written informed consent, and the Institutional Ethics Committee approved the study methodology.

Data collection

Clinical and demographic data were collected from patient medical records and supplemented by structured patient interviews where required. Annual bleeding frequency was defined as the total number of bleeding episodes over the preceding year and was obtained from documented bleeding records and treatment logs maintained at the hemophilia day care center. Both joint and non-joint bleeding episodes were included.

Assessment tools

Joint health was assessed using the HJHS version 2.1, which examines the elbows, knees, and ankles based on factors such as swelling, swelling duration, muscle atrophy, crepitus during movement, loss of flexion, loss of extension, joint pain, muscle strength, and overall gait. The highest attainable HJHS score is 124, and higher scores signify worse joint health.

The FISH, a performance-based tool that gauges independence in seven daily activities across three domains: self-care (grooming and eating, bathing and dressing), transfers (chair and floor transfers), and mobility (walking and stair climbing), was used to evaluate functional status. Higher scores indicate greater functional independence. Each action is rated on a scale of 1 to 4 based on the degree of support needed.

The HJHS was used in its original validated form in accordance with the developers’ guidelines, and the required academic licensing process was completed prior to its use. The FISH tool is available for use in clinical and academic research settings without formal permission. The study did not involve any modification, translation, or adaptation of the tools.

Statistical analysis

Continuous variables were expressed as mean ± SD, and categorical variables were presented as frequencies and percentages. Differences in mean HJHS and FISH across bleeding frequency categories were analyzed using one-way ANOVA. Post hoc pairwise comparisons were performed using the Bonferroni correction method.

The correlation between HJHS and FISH scores was assessed using Pearson’s correlation analysis. Results of ANOVA are presented using F-statistics with corresponding p-values. A p-value < 0.05 was considered statistically significant. Statistical analyses were performed using IBM SPSS Statistics for Windows, version 25.0 (released 2017; IBM Corp., Armonk, NY, USA).

Ethical clearance

The study was duly approved by the ethics committee of the institute (Biomedical Research Ethics Committee, Pandit Bhagwat Dayal Sharma Post Graduate Institute of Medical Sciences, approval number BREC/22/TH/Med.-16). All procedures undertaken in the study that involved human participants were aligned with the ethical standards established by the institutional and/or national research committee, as well as the 1964 Helsinki Declaration and its subsequent amendments or similar ethical standards.

## Results

The study included 100 patients who had been diagnosed with hemophilia. Of these, 90 patients (90%) had hemophilia A, while 10 patients (10%) had hemophilia B, indicating a predominance of hemophilia A in the study population. Regarding factor levels, the majority of participants (98%) had severe disease with factor levels <1%, whereas 2% of patients had factor levels of approximately 6%.

The distribution of affected joints demonstrated that the right knee was the most commonly involved joint, comprising 29% of cases, followed closely by the left knee at 28%. The right elbow was affected in 15% of cases, while the left elbow and right ankle were involved in 10% and 5% of cases, respectively. A small proportion of patients had involvement of the shoulder or hip joints (2% each), and one patient (1%) had no target joint involvement. The baseline demographic and clinical characteristics of the study cohort are illustrated in Table 1.

**Table 1:** Baseline demographic and clinical characteristics of persons with hemophilia (n = 100) Values are presented as numbers (percentages) for categorical variables and mean ± SD for continuous variables. FISH, Functional Independence Score in Hemophilia; HJHS, Hemophilia Joint Health Score

Demographic variables	n (%)/mean ± SD
Type of hemophilia	
Hemophilia A	90 (90%)
Hemophilia B	10 (10%)
Factor level	
Less than 1%	98 (98%)
6%	2 (2%)
Joint involvement	
Left ankle	3 (3%)
Left elbow	10 (10%)
Left hip	2 (2%)
Left knee	28 (28%)
Left shoulder	2 (2%)
Right shoulder	2 (2%)
Right ankle	5 (5%)
Right elbow	15 (15%)
Right hip	2 (2%)
Right knee	29 (29%)
No target joint	1 (1%)
Scoring system	
HJHS	15.64 ± 8.84
FISH	29.07 ± 2.73

**Figure 1:**
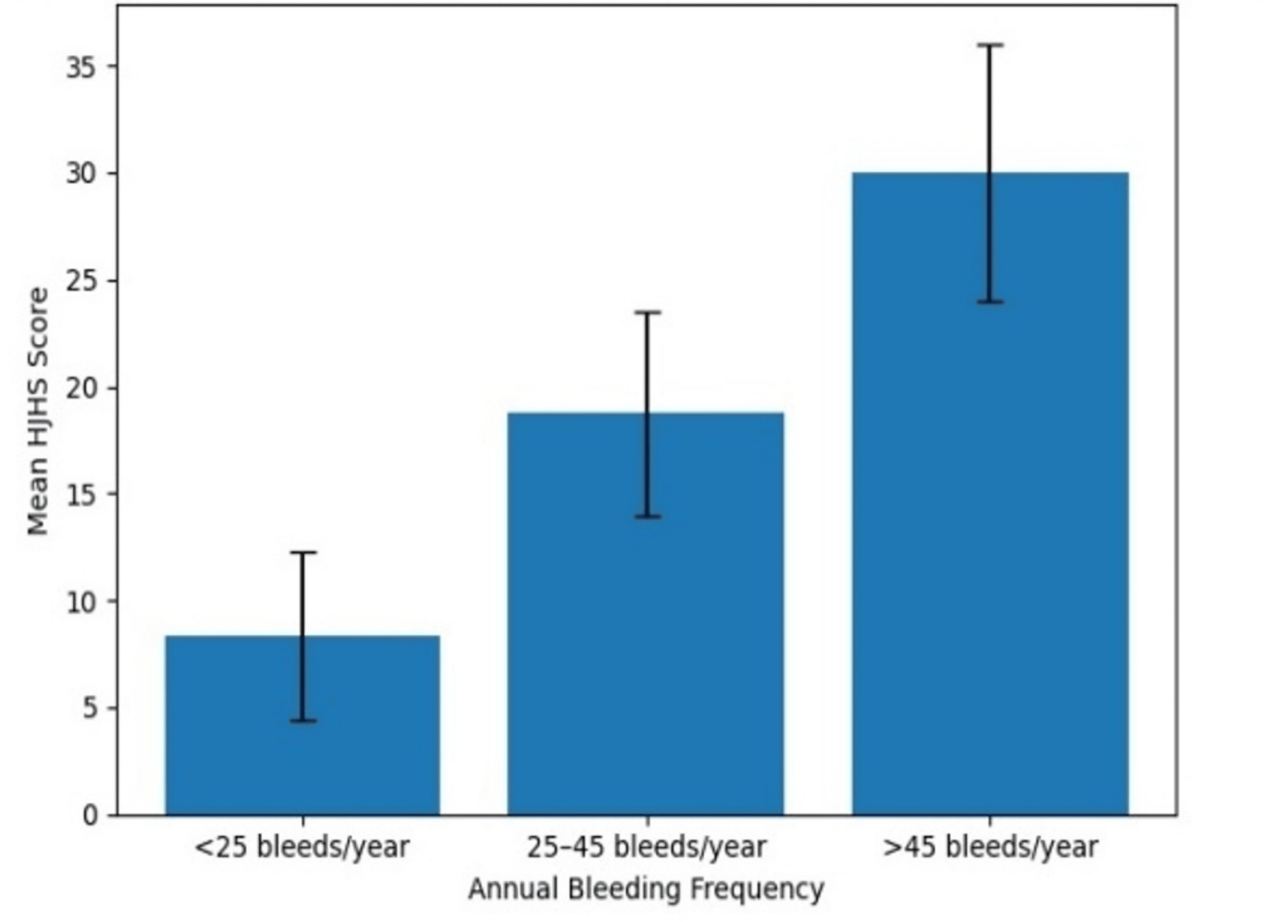
Association between annual bleeding frequency and HJHS Mean HJHS scores increased progressively with higher annual bleeding frequency, indicating worsening joint health among patients with more frequent bleeding episodes. Error bars represent SD. HJHS, Hemophilia Joint Health Score

**Figure 2:**
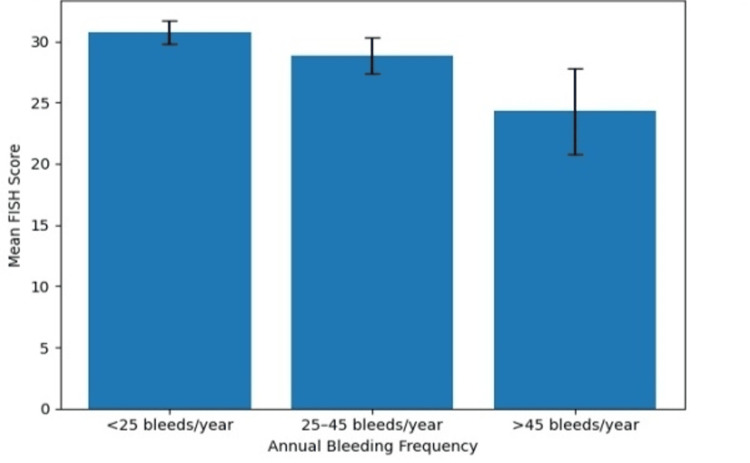
Association between annual bleeding frequency and FISH Mean FISH scores decreased with increasing annual bleeding frequency, indicating reduced functional independence among patients experiencing more frequent bleeding episodes. Error bars represent SD. FISH, Functional Independence Score in Hemophilia

The mean HJHS among study participants was 15.64 ± 8.84, while the mean FISH was 29.07 ± 2.79. The association between annual bleeding frequency and the scoring systems is presented in Table 2 and illustrated in Figure 1 and Figure 2. The mean HJHS score increased significantly with increasing number of bleeding episodes per year. Patients with <25 bleeds per year had a mean HJHS score of 8.36 ± 3.91, whereas those with 25-45 bleeds per year had a mean score of 18.73 ± 4.79. The highest mean HJHS score (30 ± 6.04) was observed among patients with >45 bleeding episodes per year, and this difference was statistically significant (p < 0.001).

**Table 2:** Association between annual bleeding frequency and HJHS and FISH Values are expressed as mean ± SD. Comparison between groups was performed using one-way ANOVA. F-statistic values and corresponding p-values are reported. ** Statistically significant (p < 0.05) FISH, Functional Independence Score in Hemophilia; HJHS, Hemophilia Joint Health Score

Scoring system	Bleeds <25/year (1)	Bleeds 25-45/year (2)	Bleeds >45/year (3)	F-value	p-value
HJHS	8.36 ± 3.91	18.73 ± 4.79	30 ± 6.04	132.62	<0.001** (1 vs 2: <0.001**, 1 vs 3: <0.001**, 2 vs 3: <0.001**)
FISH	30.78 ± 0.95	28.83 ± 1.48	24.29 ± 3.52	76.35	<0.001** (1 vs 2: <0.001**, 1 vs 3: <0.001**, 2 vs 3: <0.001**)

This trend is clearly demonstrated in Figure 1, which shows a progressive increase in HJHS scores with increasing bleeding frequency, reflecting worsening joint health. In contrast, FISH scores demonstrated a declining trend with increasing bleeding frequency. Patients with <25 bleeds per year had the highest mean FISH score (30.78 ± 0.95), which decreased to 28.83 ± 1.48 in patients with 25-45 bleeds per year and further to 24.29 ± 3.52 among those with >45 bleeding episodes per year. The differences between the groups were statistically significant (p < 0.001). As illustrated in Figure 2, functional independence progressively declined with increasing bleeding frequency.

The correlation analysis showed a strong negative relationship between HJHS and FISH scores (r = -0.905, p < 0.001), suggesting that more extensive joint damage is associated with lower levels of functional independence. Additionally, HJHS scores showed negative correlations with variables related to energy and fatigue, limitations of activities, physical health, emotional health, and overall well-being, whereas FISH scores demonstrated positive correlations with these parameters.

## Discussion

Hemophilia is associated with recurrent bleeding episodes that frequently involve the musculoskeletal system, particularly the joints. Repeated hemarthrosis results in progressive joint damage and chronic hemophilic arthropathy, which significantly affects mobility, functional independence, and overall quality of life [5,7]. The present study evaluated joint health and functional status in adult persons with hemophilia using the HJHS and the FISH, two validated tools widely used in clinical practice and research.

In the current study, hemophilia A accounted for the majority of cases, while hemophilia B constituted a smaller proportion. This distribution is consistent with global epidemiological data indicating that hemophilia A accounts for approximately 80-85% of all hemophilia cases [1,12]. This distribution is consistent with epidemiological data from India, indicating that hemophilia A represents the predominant type of hemophilia among reported cases [13]. The predominance of hemophilia A in our study, therefore, reflects the expected epidemiological pattern of the disease.

The analysis of joint involvement in the present study demonstrated that the knee joint was the most frequently affected joint, followed by the elbow and ankle joints. These findings are consistent with previous reports indicating that weight-bearing joints are particularly vulnerable to recurrent hemarthrosis due to their biomechanical load and extensive synovial vascular supply [6,8]. Repeated bleeding within these joints leads to synovial hypertrophy, inflammatory changes, and progressive cartilage destruction, resulting in the typical degenerative features of hemophilic arthropathy [7]. Similar patterns of joint involvement have been reported in multiple clinical studies evaluating musculoskeletal complications in patients with hemophilia [5].

The mean HJHS score observed in our study reflects a moderate degree of joint involvement among the study population. The HJHS has been widely used as a sensitive clinical tool for detecting early joint abnormalities and monitoring disease progression in patients with hemophilia [9]. Previous studies have demonstrated that HJHS correlates well with imaging findings and functional outcomes, making it a valuable instrument for clinical assessment and long-term follow-up [14].

The FISH was utilized to assess functional independence, evaluating the patient’s capability to execute activities of daily living, such as self-care, transfers, and mobility [11]. The relatively high mean FISH score observed in the present study suggests that many patients were able to maintain functional independence despite evidence of joint involvement. This observation may reflect improved clinical management strategies, including factor replacement therapy and supportive rehabilitation programs, which have significantly improved long-term outcomes in patients with hemophilia [15].

A notable finding of the present study was the strong negative correlation between HJHS and FISH scores, indicating that worsening joint health is closely associated with declining functional independence. Similar associations between structural joint damage and functional performance have been reported in previous studies of hemophilia, which demonstrated that higher HJHS scores are linked with reduced mobility and functional capacity [9,14,16]. Recent multicenter studies have also confirmed the validity of HJHS as a reliable tool for assessing joint health in adults with hemophilia [17].

Another important observation was the significant association between bleeding frequency and joint damage severity. This relationship is further illustrated in Figure 1 and Figure 2, where increasing bleeding frequency was associated with progressive worsening of HJHS scores and a decline in functional independence measured by FISH. Patients experiencing a higher number of bleeding episodes per year had significantly higher HJHS scores, indicating greater joint deterioration. Recurrent joint bleeding is widely recognized as the primary factor responsible for the development and progression of hemophilic arthropathy [5,7]. Each bleeding episode triggers inflammatory processes within the synovium, leading to progressive cartilage damage and irreversible joint degeneration over time [6,18].

Consistent with these findings, functional independence assessed by FISH scores showed an inverse relationship with bleeding frequency. Patients with fewer bleeding episodes demonstrated better functional ability compared with those experiencing frequent bleeding. Similar observations have been reported in previous studies, indicating that recurrent hemarthrosis contributes to reduced mobility, impaired physical functioning, and diminished quality of life in individuals with hemophilia [15,16].

Taken together, the findings of this study highlight the essential role of comprehensive assessment of joint health alongside functional status in patients diagnosed with hemophilia. The combined use of HJHS and FISH provides a practical and reliable approach for evaluating disease burden and monitoring clinical outcomes. Routine assessment using these standardized scoring systems may facilitate early identification of joint deterioration and allow timely therapeutic interventions aimed at preventing long-term disability.

Limitations

This study has certain limitations. The cross-sectional design and lack of adjustment for potential confounders preclude any causal inference between bleeding frequency and joint damage; therefore, the findings should be interpreted as associations rather than causation. The study was conducted at a single tertiary care facility, which may limit the generalizability of the findings. Additionally, imaging modalities such as MRI or USG were not used to assess structural joint changes, and the assessment relied primarily on clinical scoring systems. Larger multicenter longitudinal studies are needed to further validate these findings.

## Conclusions

Increased bleeding frequency in individuals with hemophilia is associated with worsening joint health and reduced functional independence. The strong negative correlation between HJHS and FISH highlights the close relationship between joint damage and functional impairment. Routine assessment using these standardized tools may facilitate early detection of joint deterioration and support timely interventions to preserve mobility and quality of life.
